# Quantifying Fundus Autofluorescence in Patients With Retinitis Pigmentosa

**DOI:** 10.1167/iovs.16-21302

**Published:** 2017-03

**Authors:** Kaspar Schuerch, Russell L. Woods, Winston Lee, Tobias Duncker, François C. Delori, Rando Allikmets, Stephen H. Tsang, Janet R. Sparrow

**Affiliations:** 1 Department of Ophthalmology, Columbia University, New York, New York, United States; 2 Schepens Eye Research Institute and Department of Ophthalmology, Harvard Medical School, Boston, Massachusetts, United States; 3 Department of Pathology and Cell Biology, Columbia University, New York, New York, United States

**Keywords:** retinitis pigmentosa, photoreceptor cells, optical coherence tomography, quantitative fundus autofluorescence, retinal pigment epithelium, scanning laser ophthalmoscope

## Abstract

**Purpose:**

Using quantitative fundus autofluorescence (qAF), we analyzed short-wavelength autofluorescent (SW-AF) rings in RP.

**Methods:**

Short-wavelength autofluorescent images (486 nm excitation) of 40 patients with RP (69 eyes) were acquired with a confocal scanning laser ophthalmoscope equipped with an internal fluorescent reference. Mean qAF was measured in eight preset segments (qAF_8_) and in region of interest (ROI)-qAF (200–700 μm) within and external to the borders of the rings at superior, temporal, and inferior sites relative to the ring. For both groups, qAF in patients with RP was compared to age-similar and race/ethnicity-matched healthy eyes at equivalent retinal locations.

**Results:**

In 71% of eyes of RP patients, qAF_8_ acquired internal to the inner border of the ring, was within the 95% confidence interval (CI) for healthy eyes, while in the remaining RP eyes qAF_8_ was either higher or lower than the CI. Measured external to the ring, qAF_8_ values were within the CI in 47% of RP eyes with the other eyes being higher or lower. In 28% of sites measured by ROI-qAF within the SW-AF ring, values were above the 95% CI of healthy controls. Region of interest-qAF measured just external to the ring was within the CI of healthy eyes in 74% of locations. The average local elevation in qAF within the ring was approximately 15%. In SD-OCT scans, photoreceptor-attributable reflectivity bands were thinned within and external to the ring.

**Conclusions:**

Increased fluorophore production may be a factor in the formation of the SW-AF rings in RP.

Retinitis pigmentosa comprises a group of genetically heterogeneous retinal degenerative diseases characterized by progressive loss of photoreceptor cells and constriction of the visual field over time. In patients with RP, short-wavelength fundus autofluorescence (SW-AF; excitation at 486 nm) and near-infrared fundus autofluorescence (NIR-AF; 787 nm) images often reveal a paracentral ring or arc of locally elevated AF relative to background levels in the image.^1–4^ With time the diameter of the ring progressively constricts.^5^ Outside the SW-AF ring, photoreceptor cell degeneration is evidenced by loss of the ellipsoid zone (EZ) and external limiting membrane (ELM) and thinning or absence of the reflectivity layer corresponding to outer nuclear layer (ONL) in SD-OCT scans.^6,7^ This thinning of photoreceptor-attributable reflectivity layers in SD-OCT scans also corresponds to a reduction in multi-focal ERG amplitudes.^8^ In short, the SW-AF rings are considered to reflect a transition between abnormal and normal retinal function, with function being relatively normal in retinal areas internal to the ring, reduced within the ring, and absent outside the ring.^9–13^ These rings are not genotype specific and are observed in dominant, recessive, X-linked, and syndromic RP.^6,7,14–18^

Previous analysis of SW-AF rings in images of RP patients have relied on qualitative assessment of localized AF changes within a single image.^1,6,7,14–18^ This limitation of the images arises from image processing that involves stretching of the image histogram to increase contrast. Accordingly, conventional SW-AF images display the spatial distribution of AF signal (gray levels [GLs]) at the fundus, but GL intensities within these images cannot be compared among subjects and patients or among serial images of the same individual.^19^ We recently established a method for quantitative fundus autofluorescence (qAF) wherein the SW-AF intensities in non-normalized SW-AF (without histogram stretching) are calibrated to the fluorescence of an internal reference mounted in the confocal scanning laser ophthalmoscope (cSLO) so as to compensate for variations in laser power and detector gain.^20,21^ Here we have applied this approach to the analysis of SW-AF rings in RP patients so as to better understand this SW-AF pattern in relation to disease progression.^22^ Accordingly, we sought to determine whether there is an actual increase in SW-AF intensity within the ring compared to corresponding areas in healthy retina or if there is only an apparent increase due to decreased SW-AF intensity in adjacent areas of the retina. To further characterize our cohort of RP patients, we also measured ring eccentricity and width, dynamic features previously described,^6,23,24^ and we measured outer retinal thicknesses in SD-OCT scans to correlate structural changes with aberrant SW-AF signals.

## Methods

### Patients

Forty patients (26 White, 7 Hispanic, 4 Asian, 2 Indian, 1 Black) with clinically diagnosed RP were recruited prospectively. The mean age of the cohort was 33 years (range, 10–64 years); 23 patients were female. Mean refractive error was −1.1 D (diopters) (range, −8.75 through 6 D). The Table summarizes the clinical, demographic and genetic characteristics of each patient. In addition to SW-AF imaging, clinical diagnosis of RP was based on typical fundus features, visual symptoms, family history, full-field electroretinography recorded according to standards outlined by the International Society for Clinical Electrophysiology of Vision,^25^ and SD-OCT. All patients were clinically examined by author (SHT) at the slit lamp and by fundoscopy. Exclusion criteria were substantial media opacities, corneal scars, severe vitreous floaters (visible floater shadows), pseudophakia, or insufficient pupillary dilation that would interfere with image acquisition and analysis. Systemic disease leading to retinal degeneration and medications such as chloroquine or hydroxychloroquine that affect macular function were also reasons for exclusion.

**Table:**
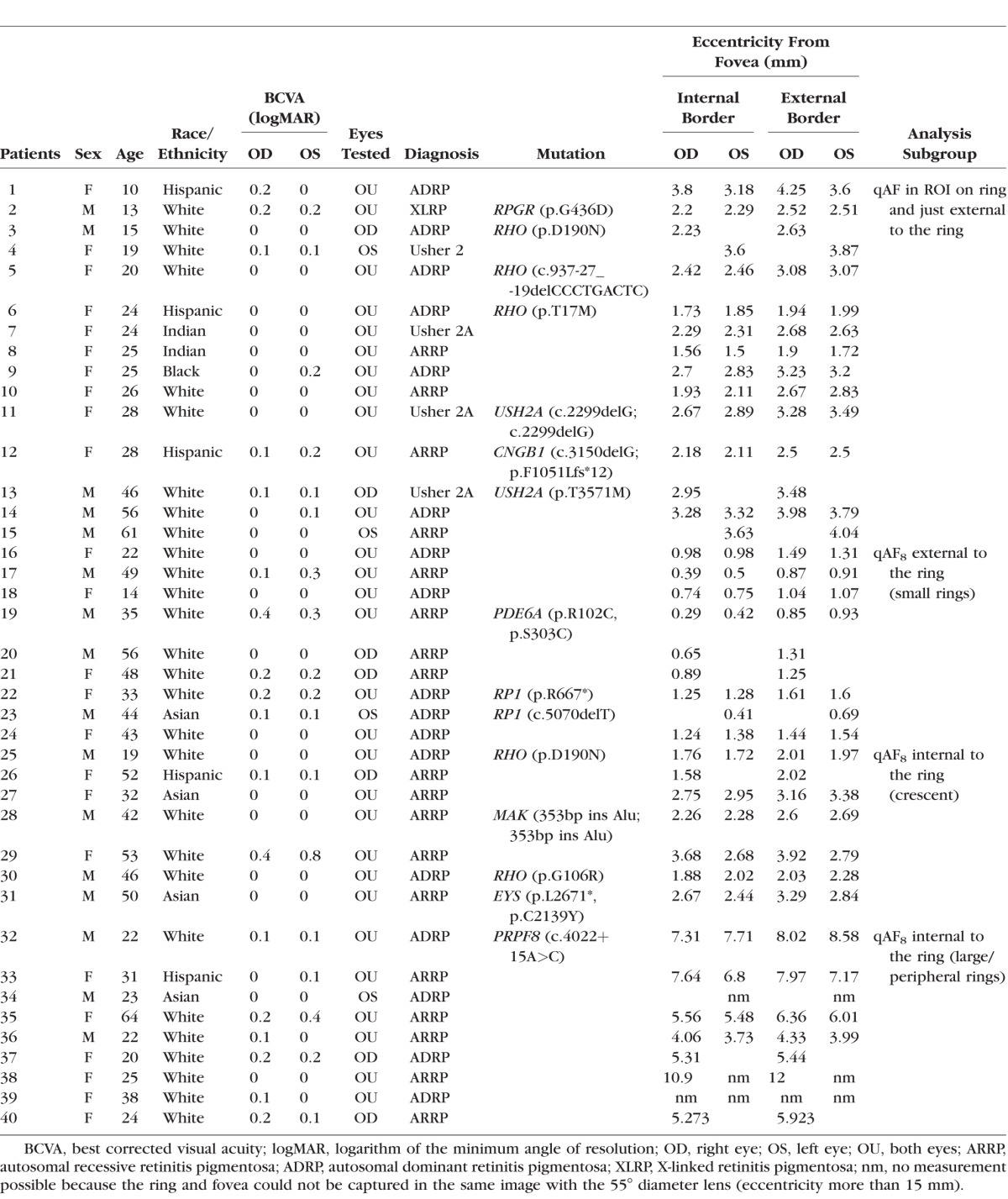
Summary of Demographic, Clinical, and Genetic Data

As inclusion criteria, all patients in the study presented with an abnormal SW-AF image characterized by a ring or arc of locally increased SW-AF. For image analysis, patients were grouped according to the size and shape of the SW-AF ring (described below). All procedures adhered to the tenets of the Declaration of Helsinki and were approved by the Institutional Review Board of Columbia University Medical Center. Written informed consent was obtained from all subjects prior to enrollment and procedures.

### Genetic Screening

Seventeen patients and family members were genetically screened by a genotyping method (APEX Arrayed Primer Extension; Asper Biotech, Tartu, Estonia) or the Retinal Dystrophy Panel (127 genes) by the Casey Eye Institute Molecular Pathology Laboratory (Oregon Health Sciences University, Portland, OR, USA) or by whole exome sequencing (Illumina HiSeq2500) at the Molecular Pathology Department of the Columbia University (New York, NY, USA).

### qAF Image Acquisition

Protocols for the acquisition of SW-AF images that meet the requirements for quantification have been described previously.^20,26,27^ Short-wavelength fundus autofluorescence images (30°; 486 nm excitation) were acquired using a cSLO (Spectralis HRA+OCT; Heidelberg Engineering, Heidelberg, Germany) modified by the insertion of an internal fluorescent reference to account for variations in laser power and detector gain. The barrier filter in the device transmitted light from 500 to 680 nm. Before image acquisition, pupils were dilated to at least 7 mm with topical 1% tropicamide and 2.5% phenylephrine. With room lights turned off, an infrared-reflectance (NIR-R) image (820 nm) was recorded first. After switching to SW-AF mode (486 nm excitation; beam power < 260 μW), the camera was slowly moved toward the patient to allow the patient to adapt to the blue light. Patients were asked to focus on the central fixation light of the device. The fundus was exposed for 20–30 seconds to bleach rhodopsin, while focus and alignment were refined to produce a uniform signal over the whole field. The detector sensitivity was adjusted so that the GLs did not exceed the linear range of the detector (GL < 175). Images were acquired in high-speed mode (8.9 frames/second) within a 30° × 30° field (768 × 768 pixels).

In a first session, two images were then recorded (each of 9–12 frames, in video format). In the majority of eyes (67/76), a second session was recorded immediately after refocusing, realigning, and adjusting the detector sensitivity. After imaging, all videos were inspected for image quality and consistency in GLs. At least four out of nine frames without localized or generalized decreased AF signal (due to eyelid interference or iris obstruction) and no large misalignment of frames (causing double images after alignment) were selected. These frames were then aligned and averaged with the system software and saved in non-normalized mode (no histogram stretching).

For each eye, two (required minimum) to four images were included in the study. Images for both eyes were available for 36 out of 40 patients. Of the 294 images available in this study, 79 (27%) were excluded due to insufficient image quality.

Retinal light exposures were below the maximum permissible levels recommended by the American National Standards Institute for healthy eyes for durations longer than 8 hours.^28^ The actual duration of the retinal exposure was always less than 90 seconds (two sessions, including bleaching); the retinal irradiance was then at least 300 times below the maximum permissible exposure.

### qAF Image Analysis

**Figure 1:**
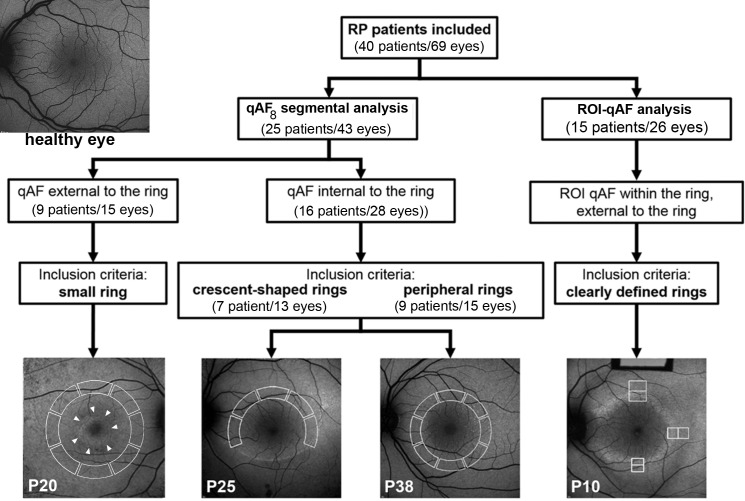
Assignment of RP patients for qAF analysis. Quantitative fundus autofluorescence external and internal to the ring was measured within circularly arranged segments (*white solid lines* in images) (segmental qAF_8_ analysis) in patients with RP characterized by small, crescent-shaped and large peripheral SW-AF rings. In the case of crescent-shaped rings, internal to the ring refers to normal-appearing retina. Region of interest-qAF (ROI-qAF) analysis included RP patients with perifoveal circular or oval SW-AF rings. Region of interest-qAF was measured using rectangular areas (*white lines* in images) located within the inner and outer borders of the ring and just external to the ring. The image, *top left*, is of a healthy eye, age 19. P, patient number.

All SW-AF images were analyzed with custom software written in Igor (WaveMetrics, Lake Oswego, OR, USA). Gray levels in the fundus image were standardized to the GL of the internal reference to generate qAF maps after accounting for the zero GL, the magnification (refraction), and ocular media absorption.^20^ Retinal vessels were excluded from the mean GL computation by histogram analysis.^21^ Because of the variability in the positions and shapes of the SW-AF rings and arcs, we assigned each patient to one of two different analytical approaches (Fig. 1).

### qAF_8_ Segmental Analysis

In 25 patients with RP (43 eyes), the configuration and size of the rings made it possible to use our standard qAF_8_ segmental analysis, together with our database of subjects with healthy eyes.^21^ In this analysis, mean qAF in each of eight circularly arranged segments at an eccentricity of approximately 7° to 9° were computed and qAFs averaged to yield a single value (qAF_8_) for each image.^21^ Segments were excluded if areas of low AF resulting from retinal degeneration or pigment migration (and identified by histogram analysis^29^) exceeded more than 50% of the area of the segment or if part of the segment was more than 15° from the center of the image.

**Figure 2:**
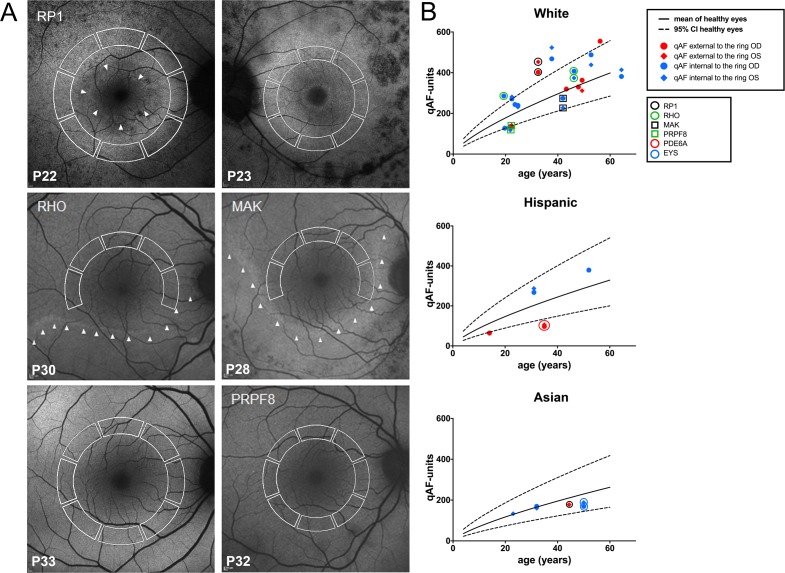
(**A**) Short-wavelength autofluorescence images of patients with RP assigned to qAF_8_ analysis. Mean GLs were recorded in eight circularly arranged segments (*white lines*) to calculate qAF_8_. The gene mutation is indicated in *upper left* of each fundus image if known. The *arrowheads* indicate the SW-AF border of the ring or crescent. P, patient number. (**B**) Mean qAF_8_ units are plotted as a function of age for each eye of patients with RP self-identified as White, Hispanic, and Asian. Symbols are color-coded for mutations and for values within and external to the ring. *RP1*, retinitis pigmentosa 1 gene; *RHO*, rhodopsin gene; *MAK*, male germ cell-associated kinase gene; *PRPF8*, precursor mRNA-processing factor 8 gene; *PDE6A*, phosphodiesterase 6A gene; *EYS*, homologue of drosophila eyes shut gene. Mean (*solid black line*) and 95% CI (*dashed line*) of healthy eyes.

Three subgroups were identified on the basis of the relative position of the AF ring and the segments (Fig. 1; Table). In 9 patients (15 eyes) having rings that were completely enclosed by the eight segments and in which the inner border of the ring was at an eccentricity of no more than 5° from the fovea, qAF_8_ was measured external to the ring. In 9 other patients (15 eyes), the ring was completely external to the eight segments, and qAF_8_ was measured internal to the ring. In these cases, the presence and extent of each ring or crescent was confirmed in wide-field SW-AF using the 55° lens. Finally, in seven patients (13 eyes), crescent-shaped SW-AF rings (or arcs) partially covered the segments or were situated at eccentricities greater than the segments (Fig. 2a). A minimum of five segments per image were required to calculate the qAF_8_. Of 320 segments, 60 (19%) were excluded due to interference by the ring. Excluded segments were always inferior, which would result in qAF values that were underestimated by approximately 4%, based on the spatial distribution over the retina in healthy eyes.^20^ Since the available segments were at smaller eccentricities than the ring or arc, for these seven patients qAF_8_ was measured internal to the ring. Mean qAF_8_ of each eye was then calculated for the available images (two to four), and qAF_8_ values acquired from the RP cohort were compared to a database of healthy eyes as described below.

### Repeatability and Concordance

For qAF_8_ measurements, between-session repeatability and concordance between eyes (of the same patient) were assessed using the Bland-Altman approach.^30^

### ROI-qAF Analysis

In 15 patients with RP (see Table) who presented with perifoveal circular or oval-shaped rings, qAF values were obtained within the ring (defined as between the inner and outer border of the ring) and immediately external to the ring for superior, temporal, and inferior sites relative to the fovea (Fig. 1). Nasal locations were not included in the analysis because the myopic crescent could interfere with the measurements. The sampling areas were defined by regions of interest (ROI-qAF) consisting of rectangular areas positioned parallel to the ring; their widths matched the apparent ring width (200–700 microns) and their length was approximately two or four times their width. The inner border of the ring was at an eccentricity of more than 5° (1.5 mm) temporally and more than 4° inferiorly and superiorly. Thus, macular pigment was not expected to influence SW-AF measurements significantly.^31^ Nonetheless, measurements taken immediately internal to the ring were only used for the analysis of the local elevation in qAF. After exclusion of vessels by the Igor software, mean qAF in the ROI rectangles was computed and the distance between the ROI and fovea was recorded. Region of interest-qAF values at each of the six sites were recorded for each image (two to four images per eye). Quantitative fundus autofluorescence values acquired from the RP cohort were compared to a database of healthy eyes as described below.

### Repeatability of ROI-qAF

To evaluate the variability introduced by the operator, ROI-qAF measurements were performed twice on all included images by the same operator (KS) within an interval of 12 weeks. A Bland-Altman analysis^30^ was performed to compare the two sets of measurements.

### Comparison to Healthy Eyes

For qAF_8_ analyses, the patients with small, large, and crescent-shaped rings were compared to our previously reported healthy-eye database recorded at the same eccentricities for 374 eyes of 277 subjects.^21^ Among the 25 RP patients, 17 were White, 4 Asian, and 4 Hispanic (30, 6, and 7 eyes, respectively), and 11 were male. For ROI-qAF analyses, each patient with RP was compared to five age-similar subjects of the same race/ethnicity within our database of healthy eyes. The ROI-qAF acquired from healthy eyes was measured at the same eccentricity (measured in pixels) as with the RP patient. Among the 15 RP patients, 9 were White, 1 Black, 2 Indian, and 3 Hispanic (14, 2, 4, and 6 eyes, respectively), and 10 were male. Of the 48 healthy eyes of control subjects, 28 were White, 11 Hispanic, 2 Black, and 7 Indian.

### Local Elevations of ROI-qAF in Association With RP Rings

The local increase in ROI-qAF within the ring in each image was characterized by the equation:

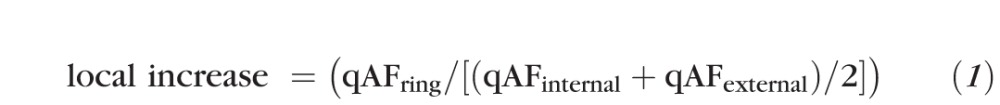



This is equivalent to the Weber contrast definition. Thus, ROI-qAF within the ring was compared to the average ROI-qAF immediately internal and external to the ring. A local increase in qAF above zero indicates that the qAF within the ring was higher than its surroundings. The local increase in qAF in the eyes of the 15 patients with RP were compared to the local increase in qAF at equivalent locations of the healthy eyes described above. Data were from three positions (internal, within, external) at three locations per image (inferior, temporal, superior) from 75 images of 26 eyes of 15 patients with RP and from 130 images of 62 eyes of 48 people with healthy eyes.

### Ring Eccentricity and Width

Eccentricity and width of the apparent ring was measured at three locations per image for all 40 patients with RP (68 eyes). At some locations, the ring could not be identified within the image, so data were available for 67 inferior, 57 temporal, and 49 superior locations. Data were available in all three locations for 48 eyes. To compare the eccentricities at the three locations, we defined relative eccentricity as the eccentricity at one location divided by the average of all three locations (available for 48 eyes).

### Outer Retinal Thickness Measurements

Horizontal 9-mm SD-OCT images through the fovea were acquired with the Spectralis; SD-OCT images were automatically registered to a simultaneously acquired SW-AF image (with correction for the magnification). Thickness measurements were performed using the caliper tool within the instrument software (Heidelberg Eye Explorer, software version 1.9.10.0). Thicknesses of two layers were measured: outer segment (OS) plus RPE complex (OS+, Bruch's membrane to EZ) and photoreceptor plus RPE complex receptor+ (REC+, Bruch's membrane to the border of the outer plexiform/inner nuclear layer [OPL/INL]).^32^ For small, peripheral and crescent-shaped SW-AF rings, these measurements were made at the midpoint of the temporal segment (eccentricity of 2.2 mm; 7.5°) used for qAF_8_ analysis. Accordingly, the location of the thickness measurement overlapped with the area used for qAF_8_ determination. For SW-AF rings assigned to the ROI-qAF analysis, thicknesses were measured at the borders of the rectangular areas used for ROI-qAF analysis. Measurements of OS+ and REC+ were compared to our healthy database.^32^

### Statistical Analysis

The data were evaluated using statistical analysis software, Prism (version 6.0c; Graphpad, La Jolla, CA, USA) and Stata (version 14; StataCorp., College Station, TX, USA). For qAF_8_ internal and external to the ring in RP patients with small, peripheral, and crescent-shaped rings, the average qAF_8_ for each of the 43 eyes (136 images, 73 sessions) was calculated. A mixed-effects model was fit to the qAF_8_ of the healthy eyes in our database adjusted for age and race/ethnicity,^21^ from which a normalized score (*z*-score) was calculated for each eye. The distribution of these *z*-scores was compared to the distribution for healthy eyes, and those outside the 95% CI were noted. For ROI-qAF within and immediately external to the ring, SW-AF images were acquired from 26 eyes of patients with RP (75 images, 40 sessions) and 62 healthy eyes (130 images). At each location (temporal, superior, and inferior) and in each eye of a patient with RP, ROI-qAF values from five age- and race-matched healthy eyes were acquired at sites equivalent to those of the patient (1163 ROI-qAF measurements from 62 eyes of 48 subjects). In addition to varying with age and race-ethnicity, qAF has been shown to vary across the retina (e.g., see Fig. 2 in Greenberg et al.^21^). A mixed-effects model was fit to the ROI-qAF data of the 62 healthy eyes that accounted for location, eccentricity, age, and race-ethnicity, from which normalized scores were calculated for each location in each eye, and those *z*-scores outside the 95% CI were noted.

To evaluate the local elevation in ROI-qAF within the SW-AF ring, a mixed-effects model was used to investigate differences between the groups (RP and healthy) and locations, with correction for ring eccentricity, eye, and age. Also, mixed-effects models were used to investigate differences in eccentricity of the ring at the three locations (temporal, superior, and inferior).

For OCT thickness measurements, 1-way ANOVA with Tukey's multiple comparison test, was used. A *P* value < 0.05 was considered as statistically significant.

## Results

Patterns of inheritance were consistent with autosomal dominant disease in 17 patients and autosomal recessive disease in 22 patients. In one patient, disease was X-linked (Table).

### SW-AF in RP

The spatial distribution of SW-AF in the fundus of a healthy eye follows a distinctive pattern. Signal is attenuated in the parafoveal region due to absorption of the excitation light by luteal macular pigment and increased optical density of melanin. Autofluorescence intensities are highest in the superotemporal quadrant and lowest in the inferonasal quadrant.^21^ In RP patients of our cohort, the spatial distribution of SW-AF was markedly changed. Readily visible SW-AF rings were located at varying eccentricities and in various shapes and sizes. Rings were symmetric in size and shape in the fellow eyes of patients assigned to the analysis, except in one patient (P3) with an irregular crescent-shaped ring (left eye); only the right eye was included in the study. The external and internal borders of the ring were discernable to varying extents. Of the three positions analyzed in relation to each ring, the temporal aspect of the ring was most likely to be clearly delineated.

### qAF_8_ Segmental Analysis of SW-AF Rings in RP

To assess qAF_8_ levels external and internal to the ring borders, we analyzed SW-AF images from 25 patients (43 eyes) (Fig. 1). For measurements taken internal to the inner border of the ring (crescent-shaped and peripheral rings; 16 patients, 13 and 15 eyes, respectively), qAF_8_ values generally fell within the 95% CI of healthy eyes (*P* > 0.05) (Fig. 2). The distribution of *z*-scores of the patients with RP was broader than that of the healthy eyes (Kolmogorov-Smirnov, *P* = 0.03), and qAF_8_ tended to be higher in the RP group (Wilcoxon-Mann-Whitney, *P* = 0.06) (Supplementary Fig. S1). In this RP subgroup (qAF_8_ internal to the inner border of the ring), two eyes had qAF_8_ below (P32; PRPF8) and six eyes had qAF_8_ above the 95% CI (P25, RHO; P36 and P39) (*P* < 0.05).

In the small ring subgroup (9 patients, 15 eyes), wherein qAF_8_ measurements were taken external to the outer borders of the rings (Fig. 2), the distribution of *z*-scores of the patients with RP was flatter than that of the healthy eyes (Kolmogorov-Smirnov, *P* = 0.05), and qAF_8_ tended to be lower in the RP group (Wilcoxon-Mann-Whitney, *P* = 0.07) (Supplementary Fig. S1). In the RP cohort, five eyes had qAF_8_ below (P16, P18, and P19 [PDE6A]) and three eyes had qAF_8_ above the 95% CI of healthy eyes (*P* < 0.05) (P20 and P22 [RP1]; Fig. 2).

Between-session repeatability of qAF_8_ was evaluated in 29 eyes. The coefficient of repeatability was ±8.7% of the mean qAF_8_, which was similar to the ±9.4% observed in healthy subjects.^21^ The coefficient of agreement for qAF_8_ between the left and right eyes was ±20.6%, a value in these severely diseased eyes that was greater than reported in healthy subjects (±15.3%).^21^ The coefficient of agreement for the center eccentricity of the rings in left and right eyes was 19%.

### ROI-qAF Analysis of SW-AF Rings in RP

**Figure 3:**
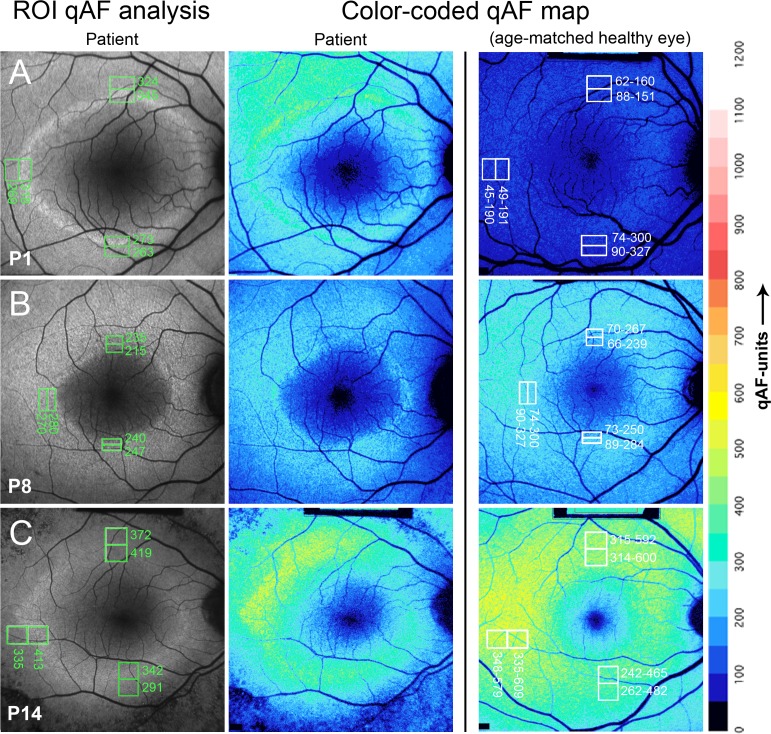
Representative SW-AF images of RP patients P1, P8, and P14. Short-wavelength fundus autofluorescence images are presented together with the positions of ROI rectangles and corresponding qAF values (column 1); color-coded maps of qAF in patients with RP (column 2); and color-coded maps of qAF of age-similar healthy eyes (column 3). Color scale of qAF units (0–1200) is provided on the right margin. P1 (**A**) represents a characteristic SW-AF ring with ROI-qAF being outside the 95% CI of healthy individuals. P8 (**B**) and P14 (**C**) show examples of patients with ROI-qAF values within the 95% CI.

**Figure 4:**
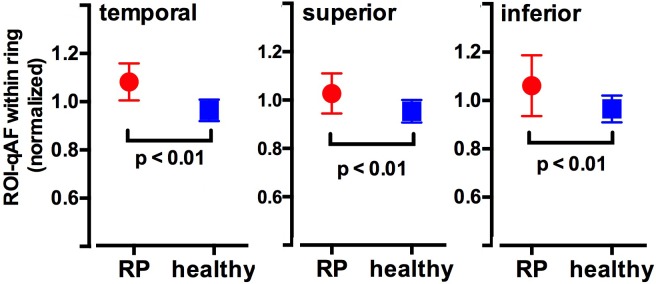
Region of interest-qAF within the ring normalized to ROI-qAF immediately external to the ring. Region of interest-qAF in healthy eyes was determined at equivalent locations. Region of interest-qAF measurements were acquired at temporal, inferior, and superior locations on the ring. Mean ± SD. *P* values determined by unpaired *t*-test.

ROI-qAF values from 15 RP patients (26 eyes) were obtained between the inner and outer borders of the SW-AF ring (within the ring) and from the region immediately external to the outer border of the ring (external to the ring) (Fig. 3). These ROI-qAF values were obtained at temporal, superior, and inferior positions of the rings for a total of 78 locations and a total of 156 ROI-qAF measurements. For an initial comparison of RP and healthy eyes, we normalized ROI-qAF within the ring to ROI-qAF external to the ring and observed that this ratio was significantly higher in eyes of RP patients than at the same positions in healthy eyes. When the ROI-qAF within the ring was normalized to ROI external to the ring, the ratio of ROI-qAF within the ring to ROI-qAF external to the ring was significantly higher in eyes of RP patients than in control subjects with healthy eyes (Fig. 4).

**Figure 5:**
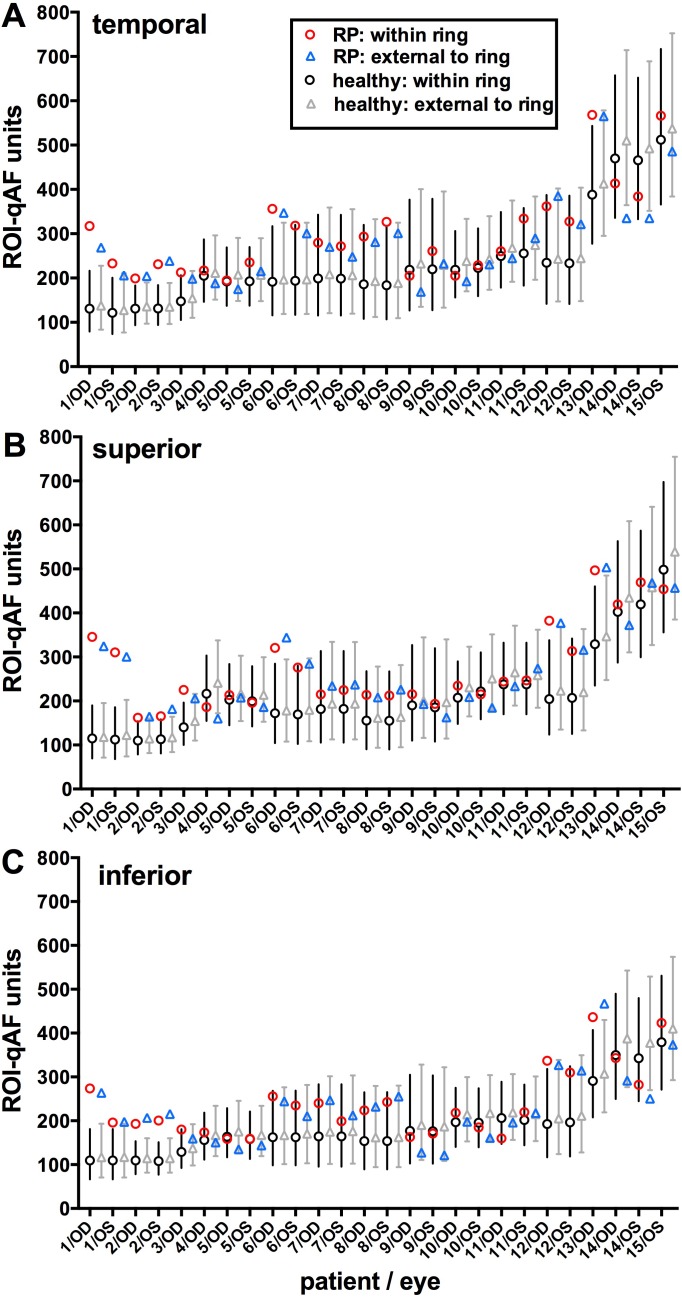
Region of interest-qAF determined for both eyes of 15 patients with RP and for healthy eyes. Patients are ranked by increasing age. Region of interest-qAF was measured at temporal (**A**), superior (**B**), and inferior (**C**) positions. For each eye, data are presented for within the ring (between inner and outer borders) and external to the ring (immediately external to the outer border). Mean ROI-qAF and CI (95%) for healthy eyes within the ring (*black circles* and *bars*) and external to the ring (*gray triangles* and *bars*) were acquired from age-similar, ethnicity-matched healthy subjects at the same fundus positions. Labels on x-axis align with RP ROI-qAF. Data plotted here are also provided in Supplementary Table S1.

Region of interest-qAF values were also compared to the CIs determined from the ROI-qAF at corresponding fundus positions of the age- and race-ethnicity–similar healthy eyes (Fig. 5; Supplementary Table S1). A mixed-effects regression model that predicted qAF was fit to the healthy-eye data, accounting for age, race-ethnicity, location, and eccentricity of the ROI. From that model, for each race-ethnicity separately, we calculated a normalized (*z*) score for each measured ROI-qAF.

For ROI of RP patients that were within the ring, 28% (22/78) had a ROI-qAF that was higher than the 95% CI determined from the healthy eyes (i.e., *z*-score > 1.96; *P* < 0.05) while the rest were within the confidence interval (*P* > 0.05). The distribution of *z*-scores of the patients with RP was flatter than that of the healthy eyes (Kolmogorov-Smirnov, *P* < 0.001) and *z*-scores were higher in the RP group (Wilcoxon-Mann-Whitney, *P* < 0.001) (Supplementary Fig. S2). In two RP patients (P1 and P2 [RPGR]), the ROI-qAF was above the CI in all three locations (temporal, superior, inferior) in both eyes.

For ROI that were external to the ring, 21% (16/78) had a ROI-qAF that was higher than the CI while 5% (4/78) had a ROI-qAF that was lower than the CI, and the rest were within the CI. The distribution of *z*-scores of the patients with RP was flatter than that of the healthy eyes (Kolmogorov-Smirnov, *P* < 0.001), and *z*-scores were higher in the RP group (Wilcoxon-Mann-Whitney, *P* = 0.0067) (Supplementary Fig. S2). Four eyes (P1 right eye; P2 both eyes [RPGR]; P13 right eye [USH2A]) exhibited ROI-qAF above the CI in all three locations.

Agreement between the first and second measurement for one image in each eye in every patient assigned to the ROI-qAF analysis was tested using the Bland-Altman analysis. The repeatability coefficient was ±5.66%, and the measurements were not different (paired *t*-test, *P* = 0.46).

### Local Elevation of ROI-qAF in Association With SW-AF Rings

**Figure 6:**
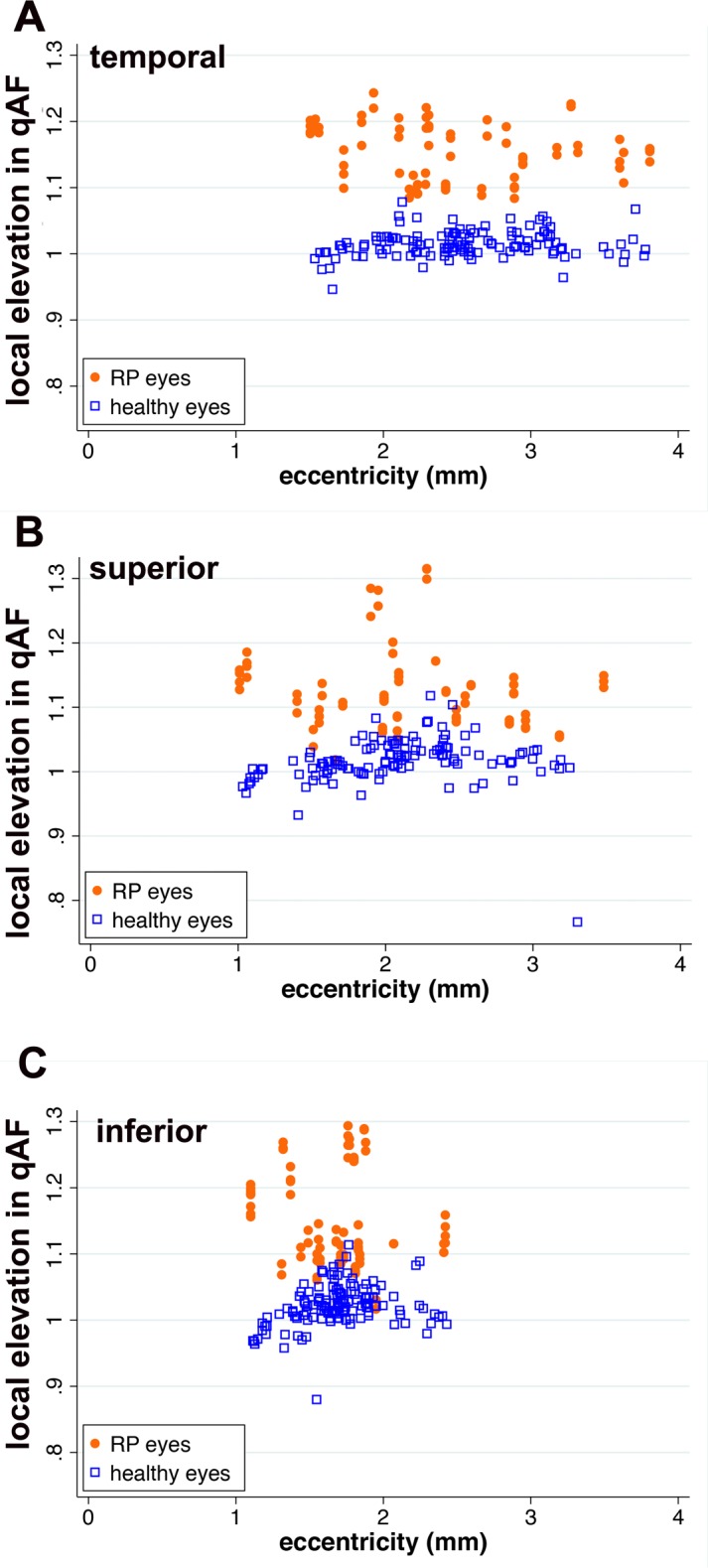
Local elevations (contrast) of ROI-qAF associated with SW-AF rings in RP patients compared to healthy eyes at the same position. Local elevation is plotted as a function of eccentricity. Each *orange dot* and *blue square* represents one measurement per image. The local elevation was calculated according to Weber contrast.

We calculated the local elevation (contrast) in ROI-qAF within the ring relative to ROI-qAF internal and external to the ring (Fig. 6). For the healthy eyes, there were no differences between locations (mixed-effects model, *z* < 1.15, *P* > 0.26), and on average, the local elevation was 0.017 (or 1.7%), which was slightly higher than zero (*z* = 1.96, *P* = 0.05) when corrected for eye (*P* = 0.12), eccentricity (*P* = 0.56), and age (*P* = 0.91). This finding indicated that ROI-qAF measured in healthy eyes at sites equivalent to ring locations in RP did not vary substantially from the ROI-qAF in adjacent regions. Conversely, the local elevation in the eyes of patients with RP was higher than among healthy eyes (*z* = 17.4, *P* < 0.001), with average local elevation being 0.16 (95% CI: 0.00–0.31) inferiorly, 0.15 (0.00–0.24) temporally, and 0.13 (0.00–0.26) superiorly. In the eyes of patients with RP, the local elevation was also lower in superior as compared to inferior (*z* = 4.46, *P* < 0.001) and temporal (*z* = 2.81, *P* = 0.005) locations, but was not different between temporal and inferior (*z* = 1.29, *P* = 0.20) sites. Local elevation also increased slightly with increasing eccentricity (*z* = 1.94, *P* = 0.05), but there was no effect of age (*z* = 0.10, *P* = 0.92).

### Ring Eccentricity and Width

**Figure 7:**
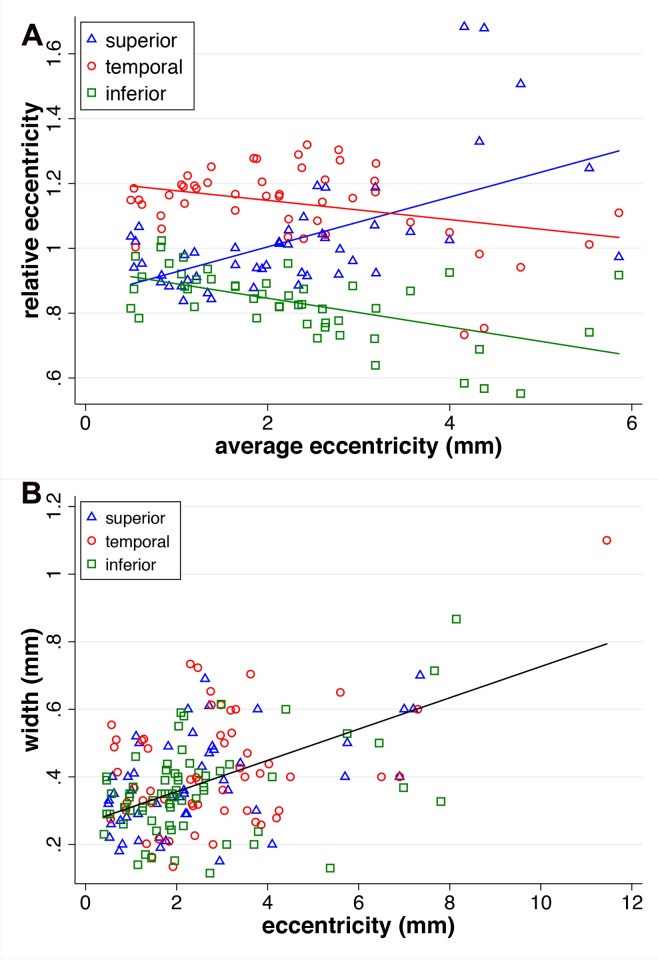
Measurements of the eccentricity of the RP rings. (**A**) Average eccentricity (mm) is plotted as a function of relative eccentricity. The latter was calculated as the eccentricity of one location divided by the eccentricities of all three locations on the same ring. (**B**) Eccentricities of the three locations on the ring are plotted against the width of the ring at the same location.

For the 68 eyes, on average, the eccentricity of the ring was less in the inferior compared to the other two locations (mixed-effects regression, *z* ≥ 5.51, *P* < 0.001), and the superior and temporal eccentricities were not different (*z* = 0.38, *P* = 0.71). Relative eccentricity (eccentricity at one location divided by the average of all three locations) varied with average eccentricity (Fig. 7). At large eccentricities, the eccentricity of the ring in the superior location was largest. As eccentricity decreased, the relative eccentricity of the superior and inferior become similar, while temporal was relatively larger at small eccentricities. The change in relative eccentricity with decreasing (“slope”) of the superior and the inferior ring was not different (*z* = 1.42, *P* = 0.16), and both were different from the temporal location (*z* ≥ 5.47, *P* < 0.001).

As shown in Figure 7, ring width decreased with decreasing ring eccentricity (mixed-effects regression, *z* = 4.00, *P* < 0.001), and that relationship did not vary among the temporal, superior, and inferior aspects of the ring (*z* ≤ 0.21; *P* ≥ 0.83).

### Retinal Thickness Measurements

**Figure 8:**
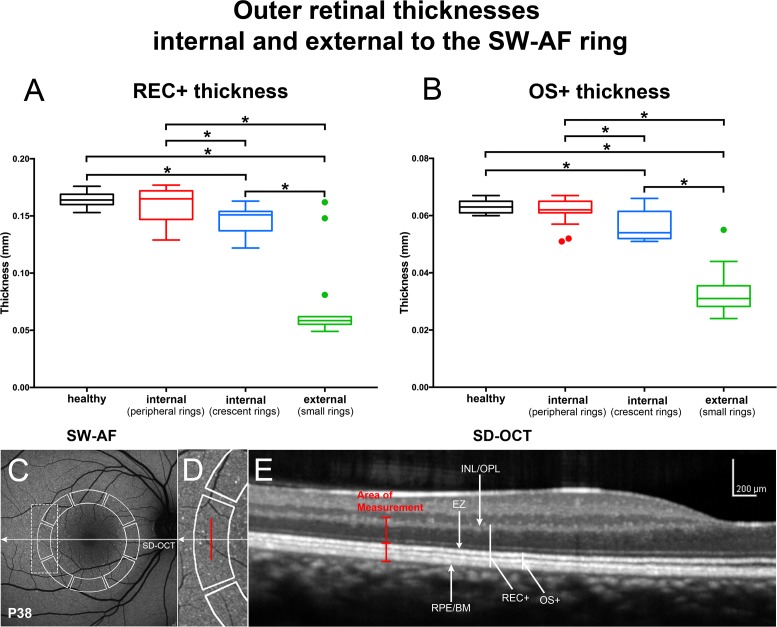
Thickness measurements in horizontal SD-OCT scans of patients analyzed using the segments employed for qAF_8_ determinations. (**A**, **B**) REC+, Bruch's membrane to the border of the outer plexiform/inner nuclear layer (INL/OPL); OS+, Bruch's membrane to EZ. Thicknesses (mm) are presented as box and whisker plots (*horizontal line*, median; *top and bottom box borders* are 25th and 75th percentiles; *horizontal lines* outside the box are minimum and maximum). In RP patients, small rings were used to measure thicknesses external to the ring; peripheral rings were used to measure thicknesses internal to the inner border of the ring. Outliers (greater than 1.5 SD from the mean) are indicated by *filled circles*. Thicknesses in RP patients are compared to a database of healthy eyes (healthy). *P < 0.01, compared to healthy eyes. (**C**–**E**) Horizontal axis of SD-OCT scan is indicated by *arrow* in corresponding SW-AF image (**C**). Measurement areas used for qAF_8_ segmental analysis are outlined in SW-AF image (*solid white line*). Area outlined by *dashed rectangle* in SW-AF image in **C** is enlarged in **D**. *Vertical red line* in **D** and **E** marks the position of thickness measurements acquired at midpoint of the temporal segment. RPE/BM, retinal pigment epithelium/Bruch's membrane.

For the subgroup of eyes having large peripheral rings (where measurements were taken internal to the inner border of the ring), thicknesses of both the OS+ and the REC+ were within the normal range. In the subgroup of crescent-shaped rings, measurements were also acquired internal to the inner border of the ring and revealed that both the mean OS+ and the REC+ thickness values were significantly thinner than the healthy eyes (*P* < 0.05). Measurements (statistically significant) external to the outer border of ring (small ring subgroup) revealed thicknesses that were thinner than healthy eyes (Fig. 8).

**Figure 9:**
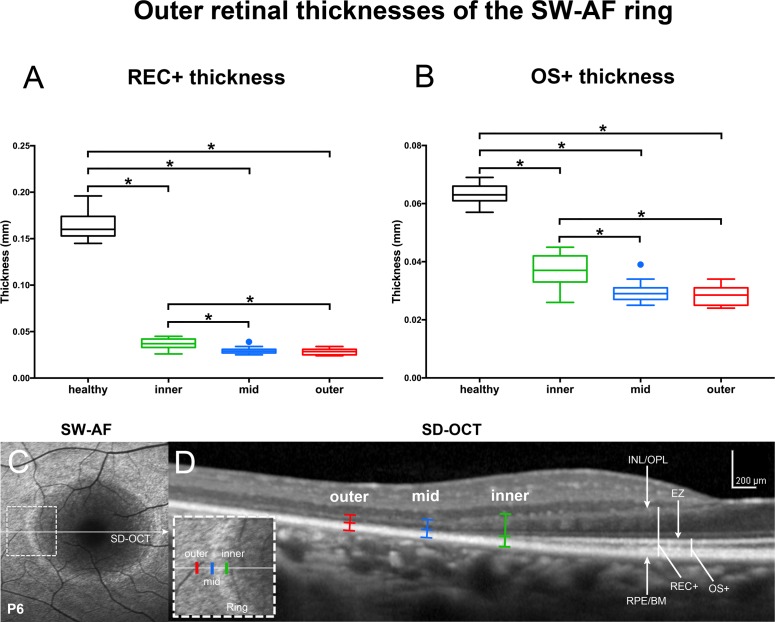
Thickness measurements in horizontal SD-OCT scans of RP patients measured with the ROI-qAF analysis. (**A**, **B**) REC+, Bruch's membrane to the border of the INL/OPL; OS+, Bruch's membrane to EZ. Thicknesses (mm) presented as box and whisker plots were acquired at temporal locations of the rings used for ROI-qAF analysis and included the inner border of the ring (inner), within the ring (mid), and immediately external to the outer border of the ring (outer). Thicknesses are compared to a database of healthy eyes (healthy). *P < 0.01, compared to healthy eyes. (**C**, **D**) Representative SW-AF and SD-OCT images employed for thickness measurements. Horizontal axis of SD-OCT scan (**D**) is indicated by *arrow* in corresponding SW-AF image (**C**). Area indicated by *dashed rectangle* in SW-AF image (**C**) is enlarged in inset of D. Outer, mid, and inner positions are indicated in SD-OCT scan (**D**). RPE/BM, retinal pigment epithelium/Bruch's membrane.

Analysis of SD-OCT images acquired from patients assigned to ROI-qAF analysis revealed REC+ thickness at all three measurement sites to be statistically lower compared to healthy eyes. Outer segment-positive measurements performed at the inner border of the ring, where the EZ is no longer intact, revealed thicknesses that were higher than at the outer border and outside of the ring. The ANOVA analysis for multiple comparisons revealed that the measurements made at the inner border of the ring were statistically higher (*P* < 0.001) than the ones at the outer border of the ring and outside the ring (Fig. 9).

## Discussion

The SW-AF rings in fundus images of patients with RP are visible with both SW-AF (486 nm) and NIR-AF (787 nm) excitation.^33,34^ Autofluorescence rings are also visible in patients with cone-rod dystrophy, and in these patients an expansion of the ring with time can be observed.^16,17^ Conversely, in patients with RP, the diameter of the ring progressively constricts.^35^ Functional analyses indicate that at the position of the AF rings visible in fundus SW-AF images, visual sensitivity is reduced.^9,10^ Moreover, in OCT scans the internal edge of the ring corresponds to the eccentric position at which the hyperreflective band attributable to the inner segment EZ is no longer intact.^11,12,18,24^ Taken together, these observations indicate that photoreceptor cells within the ring (i.e., between the inner and outer borders of the ring) are impaired.

By applying qAF protocols to the analysis of the RP rings by ROI-qAF analysis, we have shown here that in some patients (28% of measured ROI) the SW-AF of the ring reflects an actual increase in SW-AF intensity within the ring relative to corresponding areas in healthy retina. In some patients, including P13 with USH2A mutation and P1 and P2, ROI-qAF measurements revealed elevations both within the ring and external to the ring as compared to healthy eyes (Fig. 5). As with patients that do not exhibit increased qAF values, we expect that the patterns of intensities depend on the stage of disease. The qAF images we acquired provided data at a single stage of the disease while qAF can be expected to vary with disease progression. Studies in the future could relate changes in qAF to disease duration and age and could determine whether high qAF levels within the ring reflect the stage of disease.

To analyze areas larger than the ROI and to measure SW-AF intensities internal and external to the borders of the ring, we employed our previously described segment analysis (qAF_8_)^29^ and assigned rings to small, crescent-shaped or peripheral subgroups. As expected, measurements performed internal to the inner border of the ring produced qAF_8_ values that were not significantly different from healthy eyes. Here, OCT thicknesses were also in the normal range. Conversely, mean OCT thickness measurements acquired internal to the ring (in crescent-shaped rings) were statistically thinner than in healthy eyes although qAF_8_ levels at this location were usually within the 95% CI of healthy eyes. In the small ring subgroup where we measured qAF_8_ external to the outer border of the ring, qAF values were higher than the 95% CI in some cases.

External to the outer border of the SW-AF ring, the signal in NIR-AF images is diminished,^2^ probably due to RPE thinning and reduced melanin pigmentation in RPE due to proliferation-related melanin dilution. Peripheral-ward migration of the proliferating cells culminates in bone spicule pigmentation.^36^ Retinal pigment epithelium thinning external to the ring, together with severe photoreceptor cell degeneration and photodegradation of the fluorophores,^37,38^ likely accounts for the reduced SW-AF signal that characterized some of the RP eyes in this study. The outer border of the ring in NIR-AF images also corresponds to the termination of the EZ band in SD-OCT images.^2^ In SW-AF images, the position of EZ termination is interior to the outer border of the ring.^2^ If the presence of the SW-AF ring was due to increased transmission through thinned neural retina, then the outer border of the SW-AF ring should correspond to the position of EZ termination.

We have given consideration to factors that could account for the visibility of SW-AF rings in RP. Foremost is an increase in the formation of bisretinoid lipofuscin resulting in an increase in SW-AF of approximately 15%. Since our imaging protocol includes photopigment bleaching before image acquisition, it is unlikely that photopigment-related unmasking of RPE SW-AF accounts for the presence of the SW-AF rings. The abnormal SW-AF from the ring cannot be attributed to accelerated phagocytosis of photoreceptor OSs, as has been suggested,^18,22,35^ since, as shown in studies of the Royal College of Surgeons rat,^39^ bisretinoid fluorophore formation occurs in photoreceptor cells prior to OS shedding and subsequent phagocytosis. By analyzing SD-OCT images, we have shown that within SW-AF rings (i.e., between the inner and outer borders), the total thickness of photoreceptor-attributable reflectivity bands in all RP eyes of our cohort was significantly thinner than in healthy subjects. If thinning of the overlying neural retina with creation of a window defect was an explanation for the SW-AF rings in RP, one would expect AF within the ring to be consistently higher in all RP eyes, but this was not the case. By examining the SD-OCT images of patients with SW-AF rings measured with ROI-qAF, we observed increased transmission to the choroid at locations external to the ring, but not within the ring (not shown).

This study has limitations. The cohort was relatively small, and the genotypes were heterogeneous. Mutations were known in only 15 of 40 patients (17 patients were screened, two results were inconclusive). Although this was a cross-sectional study, the differences in ring eccentricity detected here are consistent with a previous report indicating that changes in the horizontal and vertical ring diameters, as a function of time, occur independently of one another.^5^ Short-wavelength autofluorescence rings vary in terms of the shape and position in the fundus, and there can be centrifugal and centripetal components to disease progression depending on the stage of the disease.^40,41^ These changes in the direction of disease evolution can complicate the assignment of areas outside and inside the ring and may have influenced the qAF_8_ segment analysis. Opacities of the lens and vitreous are more common among patients with RP than among healthy subjects, even at younger ages. Although we excluded images associated with obvious opacities, some of the eyes in our study may have had floaters or minor lens opacities that reduced qAF values. Accordingly, the qAF levels of RP patients in our results might be underestimated. Furthermore, the ROI method used to quantify SW-AF within the ring only allowed us to analyze a limited area and not the ring in its entirety. Since the ROI rectangles in the control eye and eye of the RP patient were recorded as pixel coordinates and fellow eyes had different scaling factors (micrometer/pixel), the location defined only by pixel eccentricity or pixel coordinates may not generate precisely the same location in the retina of both eyes.

Short-wavelength autofluorescence rings are dynamic features of the RP fundus. The RP patients in our cohort also differed in terms of age and genotype and, importantly, represented various stages of the disease process. Nevertheless, in the presence of this heterogeneity we observed a within-ring increase in qAF in 28% of measured ROI-qAF. Whether all patients at some period in the disease process would express a similar increase is not known but is worth consideration. Since SD-OCT and functional studies indicate that photoreceptor cells within the ring are degenerating,^42^ we suggest that the increase in qAF within the ring relative to qAF at the same position in normal eyes may indicate that the process of photoreceptor cell degeneration per se leads to increased formation of lipofuscin, measured as increased fundus SW-AF. We have previously presented data indicating that disease-related SW-AF patterns are indicative of an accelerated lipofuscin synthetic pathway initiated in disabled photoreceptor cells unable to detoxify excess retinaldehyde of the visual cycle.^43,44^ This explanation may also account for increased qAF external to the ring, with the stage of progression being a determinant of the SW-AF levels. With time, bisretinoid autofluorescence bleaching^45^ and resolution of degenerating debris would likely lead to a reduction in SW-AF intensity. It is worth noting that multimodal imaging of SW-AF flecks in the fundus of patients with recessive Strgardt disease (STGD1) has shown that at positions of hyperautofluorescent flecks in SW-AF images, NIR-AF imaging reveals an absence of RPE, while in SD-OCT images flecks correspond to hyperreflective deposits indicative of ongoing photoreceptor cell degeneration.^46^ One explanation for this otherwise anomalous finding is that the bright SW-AF signal of flecks originates not from RPE but from augmented lipofuscin formation in degenerating photoreceptor cells.

## Supplementary Material

Supplement 1

Supplement 2

Supplement 3
