# Supplementary material for: Quantifying Fundus Autofluorescence in Patients With Retinitis Pigmentosa
Source: Invest Ophthalmol Vis Sci. 2017 Mar;58(3):1843–55. doi: 10.1167/iovs.16-21302 (PMC5377994; doi:10.1167/iovs.16-21302)
Supplement: Supplement 1 [file iovs-58-03-38_s01.pdf]

Table S1. Region of interest-quantitative fundus autfluorecence (ROI-qAF): Within the autofluorescent ring and just exterior to the ring.

	within					*exterior*				
Temporal	RP	mean	U 95%	L 95%	Outside CL	RP	mean	U 95%	L 95%	Outside CL
1 OD	317.3	130.8	216.2	79.1	*	268.9	137.7	227.5	83.3	*
1 OS	233.0	121.4	200.6	73.5	*	206.0	127.6	210.9	77.2	
2 OD	199.0	130.8	183.2	93.5	*	204.5	135.8	190.0	97.0	*
2 OS	231.0	131.4	184.0	93.9	*	238.5	135.0	188.9	96.4	*
3 OD	212.5	147.3	206.2	105.2	*	198.4	154.3	216.0	110.2	
4 OD	217.1	205.1	287.1	146.5		188.3	211.6	296.2	151.2	
5 OD	194.4	192.3	269.2	137.4		174.9	207.6	290.7	148.3	
5 OS	235.2	192.7	269.7	137.6		215.1	207.0	289.8	147.9	
6 OD	356.0	191.7	316.8	116.0	*	347.2	196.5	324.7	118.9	*
6 OS	317.9	193.6	319.9	117.1		300.6	196.7	325.0	119.0	
7 OD	279.7	199.1	343.1	115.5		270.2	208.4	359.1	120.9	
7 OS	271.6	198.7	342.4	115.3		248.2	206.2	355.4	119.7	
8 OD	293.6	185.7	320.1	107.8		281.2	193.0	332.7	112.0	
8 OS	326.7	183.8	316.6	106.6	*	301.2	188.5	324.8	109.4	
9 OD	205.0	218.6	376.7	126.9		169.0	232.4	400.5	134.9	
9 OS	260.9	219.9	378.9	127.6		232.5	229.4	395.3	133.1	
10 OD	205.0	218.6	306.0	156.2		192.5	238.2	333.5	170.2	
10 OS	228.7	222.9	312.0	159.2		230.6	242.6	339.6	173.3	
11 OD	260.5	249.4	349.1	178.2		244.5	268.0	375.1	191.5	
11 OS	334.3	255.7	358.0	182.7		290.0	274.3	384.0	195.9	
12 OD	361.9	234.3	387.2	141.8		385.0	243.3	402.1	147.2	
12 OS	327.5	233.5	385.8	141.3		321.5	244.5	404.0	147.9	
13 OD	568.1	388.2	543.3	277.3	*	565.1	413.0	578.2	295.1	
14 OD	413.5	469.8	657.6	335.6		334.9	510.2	714.2	364.5	†
14 OS	383.9	465.9	652.1	332.8		335.0	492.3	689.1	351.7	†
15 OS	566.3	512.2	717.0	365.9		485.5	537.4	752.3	383.9	

Superior	RP	mean	U 95%	L 95%	Outside CL	RP	mean	U 95%	L 95%	Outside CL
1 OD	345.5	114.9	190.0	69.6	*	324.6	118.1	195.2	71.5	*
1 OS	310.5	112.4	185.7	68.0	*	300.5	122.5	202.5	74.1	*
2 OD	162.0	110.1	154.1	78.7	*	164.5	114.6	160.4	81.8	*
2 OS	165.5	113.0	158.2	80.7	*	181.5	117.4	164.3	83.9	*
3 OD	225.0	140.3	196.3	100.2	*	206.0	154.3	216.0	110.2	
4 OD	186.0	216.5	303.1	154.7		160.0	241.2	337.6	172.3	†
5 OD	213.5	202.8	283.8	144.9		207.6	216.3	302.8	154.5	
5 OS	196.0	199.3	279.0	142.4		186.0	213.8	299.3	152.7	
6 OD	320.5	172.2	284.5	104.2	*	344.2	178.2	294.4	107.8	*
6 OS	275.9	169.6	280.2	102.6		284.7	179.6	296.8	108.7	

7 OD	214.8	181.8	313.3	105.5		234.4	193.9	334.2	112.6	
7 OS	224.9	182.0	313.5	105.6		237.4	193.8	333.9	112.5	
8 OD	213.8	155.4	267.8	90.2		208.0	161.4	278.1	93.6	
8 OS	212.2	155.1	267.3	90.0		225.9	163.4	281.6	94.8	
9 OD	215.5	189.8	327.1	110.2		192.9	200.0	344.6	116.1	
9 OS	193.5	185.6	319.9	107.7		162.5	197.3	340.0	114.5	
10 OD	234.5	207.3	290.1	148.1		209.0	230.9	323.2	164.9	
10 OS	214.9	221.7	310.4	158.4		184.6	251.0	351.4	179.3	
11 OD	244.0	237.6	332.5	169.7		233.5	265.1	371.1	189.4	
11 OS	246.9	237.6	332.5	169.7		274.1	258.5	361.9	184.7	
12 OD	382.5	204.5	338.0	123.8	*	377.0	223.4	369.2	135.2	*
12 OS	313.5	207.0	342.0	125.2		316.0	220.0	363.6	133.1	
13 OD	496.6	329.0	460.5	235.0	*	503.5	346.6	485.2	247.6	*
14 OD	419.4	402.4	563.2	287.4		372.5	434.7	608.5	310.6	
14 OS	469.5	419.4	587.0	299.6		468.5	458.0	641.1	327.2	
15 OS	454.0	498.2	697.4	355.9		457.0	539.3	754.9	385.2	
Inferior	RP	mean	U 95%	L 95%	Outside CL	RP	mean	U 95%	L 95%	Outside CL
1 OD	273.7	109.7	181.2	66.4	*	263.7	116.9	193.2	70.7	*
1 OS	196.0	109.6	181.1	66.3	*	197.5	117.0	193.3	70.8	*
2 OD	193.0	109.6	153.4	78.3	*	206.5	114.3	160.1	81.7	*
2 OS	200.5	108.0	151.1	77.1	*	215.4	114.4	160.2	81.8	*
3 OD	179.9	129.1	180.7	92.2		159.6	137.3	192.2	98.1	
4 OD	173.5	156.0	218.4	111.4		150.7	167.1	234.0	119.4	
5 OD	158.7	163.5	228.9	116.8		135.2	175.5	245.7	125.4	
5 OS	159.2	158.1	221.3	112.9		143.9	167.3	234.2	119.5	
6 OD	255.7	162.5	268.5	98.3		244.2	167.1	276.2	101.1	
6 OS	235.0	162.6	268.7	98.4		210.3	170.6	281.9	103.2	
7 OD	240.0	164.5	283.4	95.4		247.0	175.0	301.5	101.5	
7 OS	199.0	164.5	283.4	95.4		212.2	176.2	303.6	102.3	
8 OD	223.8	153.8	265.0	89.3		232.0	162.2	279.5	94.1	
8 OS	242.9	153.9	265.3	89.3		255.4	162.6	280.2	94.4	
9 OD	163.0	177.1	305.1	102.8		127.5	190.4	328.1	110.5	
9 OS	170.9	176.0	303.3	102.1		121.3	186.9	322.1	108.5	
10 OD	218.0	196.6	275.2	140.5		197.9	214.1	299.6	152.9	
10 OS	185.0	195.8	274.0	139.8		161.0	217.3	304.2	155.2	
11 OD	159.8	206.4	288.9	147.4		195.9	218.8	306.3	156.3	
11 OS	219.8	201.9	282.6	144.2		217.6	215.2	301.2	153.7	
12 OD	337.0	192.7	318.4	116.6	*	327.0	205.0	338.8	124.0	
12 OS	310.0	196.3	324.4	118.8		314.4	211.4	349.4	127.9	
13 OD	436.2	290.8	407.0	207.7	*	467.1	307.2	430.0	219.4	*
14 OD	342.9	349.8	489.7	249.9		291.5	387.7	542.6	276.9	
14 OS	282.0	342.8	479.8	244.9		250.5	377.8	528.9	269.9	†

15 OS	423.0	379.3	530.9	270.9		373.5	410.0	573.9	292.9

RP, average ROI-qAF values for patients with retintitis pigmentosa; mean, average predicted ROI-qAF values for healthy eyes; U 95%, average predicted upper 95% confidence interval for healthy eyes; L 95%, average predicted lower 95% confidence interval for healthy eyes. CL, confidence limits. Predicted ROI-qAF values for healthy eyes were adjusted for age, race/ethnicity and eccentricity. * higher than the U 95%; † lower than L 95%.
