# Supplementary material for: Quantifying Fundus Autofluorescence in Patients With Retinitis Pigmentosa
Source: Invest Ophthalmol Vis Sci. 2017 Mar;58(3):1843–55. doi: 10.1167/iovs.16-21302 (PMC5377994; doi:10.1167/iovs.16-21302)
Supplement: Supplement 2 [file iovs-58-03-38_s02.pdf]

Supplementary Figure S1

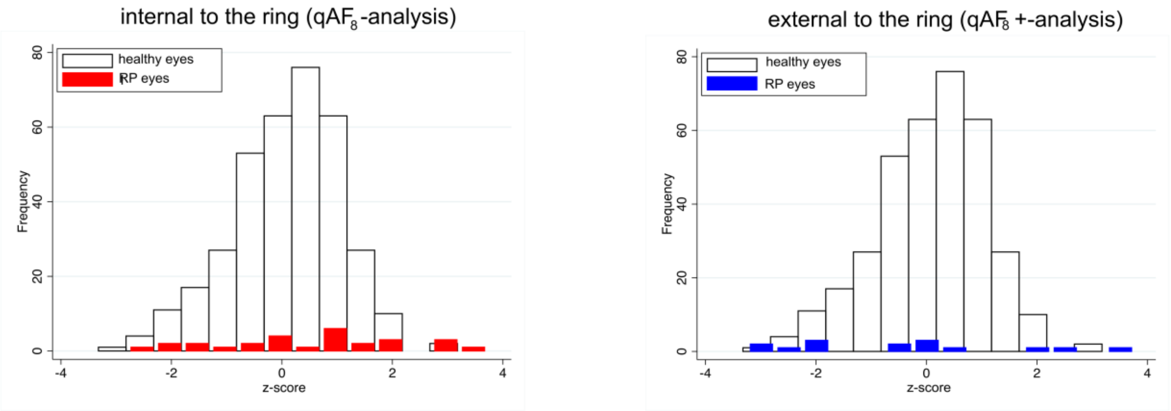

Supplementary Figure S1. z-score distribution in qAF_8 analysis in SW-AF images. Internal to the ring represents the z-score distribution of qAF_8 values of healthy and RP eyes internal to the inner border of the ring (measurements taken in patients with peripheral and crescent shaped rings) and external to the ring (acquired in patients with small rings).
