# Supplementary material for: Quantifying Fundus Autofluorescence in Patients With Retinitis Pigmentosa
Source: Invest Ophthalmol Vis Sci. 2017 Mar;58(3):1843–55. doi: 10.1167/iovs.16-21302 (PMC5377994; doi:10.1167/iovs.16-21302)
Supplement: Supplement 3 [file iovs-58-03-38_s03.pdf]

Supplementary Figure S2

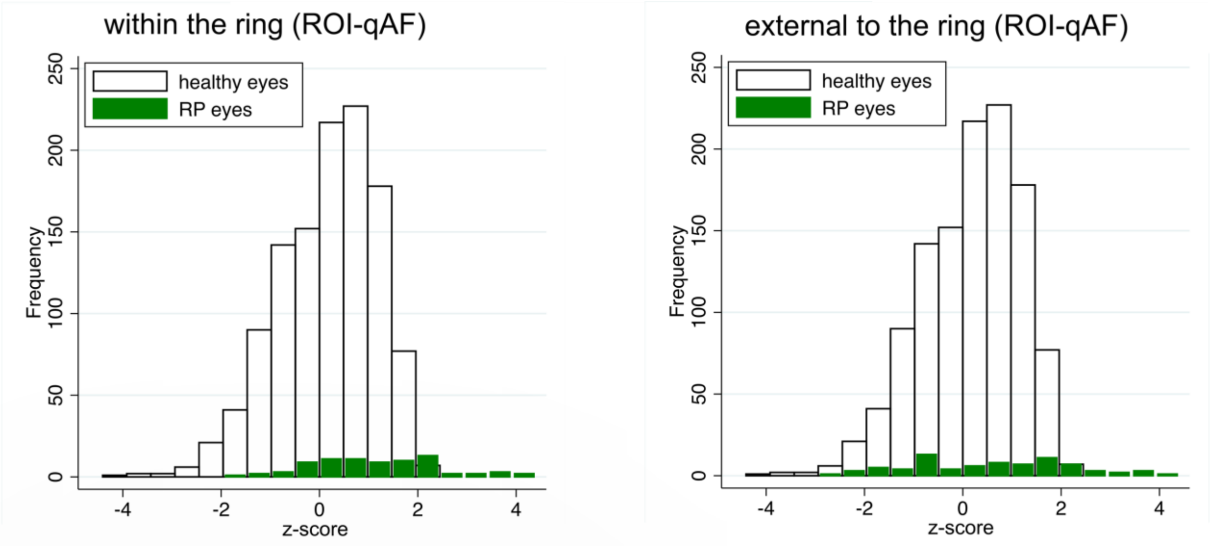

Supplementary Figure S2. z-score distribution of ROI-qAF analysis. Within the ring shows the z-score distribution of ROI-qAF values of RP eyes (measurements taken within the inner and outer border of the SW-AF ring) and corresponding ROI-qAF values of healthy eyes. Measurements external to the ring represent z-scores of ROI-qAF values acquired immediately external to the outer SW-AF border of the ring in patients with small rings and in corresponding areas of healthy individuals.
